# 9.L. Workshop: A motivated, healthy and high-performing workforce: Innovation in health workforce research in Europe

**DOI:** 10.1093/eurpub/ckac129.591

**Published:** 2022-10-25

**Authors:** Organised by: Technische Universität Berlin, Chair persons: Reinhard Busse (Germany)

## Abstract

**Background:**

Hospitals and their health care staff were severely impacted by the Covid-19 pandemic. Many health professionals faced high levels of stress and burn-out due to unfavorable work environments, limited staffing and highly pressured workloads. Yet, even prior to the pandemic, health professionals in many countries faced challenging work environments with often limited resources and support. At the same time, the roles of health professionals are changing across Europe, with new roles, task-shifting and sharing happening in teams, also driven by the Covid-19 pandemic. From a health system and management perspective, recruitment and retention is critical to ensure that there is a sufficient and well-educated health workforce capable to perform high-quality care. Multiple innovations have been implemented across Europe, these will be presented and discussed with a view towards evaluating complex interventions, sustaining innovations, ensuring the scalability and transferability of proven interventions for the benefit of health professionals and wider society.

**Objectives:**

This workshop will address innovative strategies at the micro (team level), meso (healthcare setting) and macro level (policy) to ensure that health professionals are motivated, perform high-quality care in teams and are sufficiently supported by enabling work environments and recruitment and retention policies. Innovations to improve work environments, task-shifting as well as recruitment and retention will be discussed, based on the findings from four EU-funded research projects: Magnet4Europe, H-Work, TASHI and METEOR. The format is a ‘round table’ with 5 panel discussants. The Magnet4Europe project focuses on the reorganization of hospital work environments for nurses and physicians in hospitals in five European countries. Innovative elements include a system-, inter-professional, organization-wide change management, structural empowerment of clinical staff and one-on-one twinning of European hospitals with U.S. twinning partners.

**Innovations in the EU projects:**

The H- WORK project aims to design, implement and validate psychosocial interventions to promote mental health and well-being in SMEs and the public sector. Innovative elements are the evaluation of interventions at multiple levels (individual, team, manager and organisational level) and the development of the H-Work innovation platform with toolkits, policy briefs and novel digital solutions. The TaSHI project provides a novel and up-to-date knowledge on task shifting and transferability of good practices in implementation. Through a cross-national and -sectoral perspective, the focus is on task shifting as an innovation towards more effective organisation of care and workforce, to improve efficient and sustainable systems. METEOR aims to enhance scientific knowledge on the predictors of job retention for healthcare workers and develops evidence-based policy recommendations through stakeholder engagement and co-creation workshops.

**Key messages:**

• Innovations to improve work environments include system-wide, integrated approaches spanning individual, team, management and organisational levels.

• Elements of effective implementation of innovations are effective leadership at highest level, empowerment, evidence-informed action and tailored strategies on recruitment and retention of staff.

**Speakers/Panellists:**

**Walter Sermeus**

KU Leuven, Leuven, Belgium

**Ronald Batenburg**

NIVEL, Utrecht, Netherlands

**Lode Godderis**

KULeuven - IDEWE, Leuven, Belgium

**Rudolf Kubik**

Qedgroup, Prague, Czechia

**Claudia Maier**

Department of Healthcare Management, Technische Universität Berlin, Berlin, Germany

